# Devastating Pneumococcal Arthritis of the Shoulder After Two Corticosteroid Injections

**DOI:** 10.7759/cureus.21006

**Published:** 2022-01-07

**Authors:** Benjamin Kraler, Philipp Bissig, Richard W Nyffeler

**Affiliations:** 1 Orthopaedics and Traumatology, Orthopaedic Hospital Sonnenhof, Bern, CHE; 2 Orthopaedics and Traumatology, Aarberg Hospital, Aarberg, CHE

**Keywords:** adhesive capsulitis, frozen shoulder, corticosteroid injections, corticosteroids, streptococcus pneumonia, pneumococcal arthritis, septic arthritis

## Abstract

A 36-year-old man was treated with two intraarticular corticoid injections for intense pain and severely decreased range of motion of his left shoulder. After the second injection, he came back with fulminant arthritis. Microbiological examination revealed streptococcus pneumoniae. Open debridement, long-term antibiotics, and total shoulder replacement were necessary to restore acceptable shoulder function. The fulminant course with rapid destruction of the joint illustrates the risks of intraarticular corticoid injections. This case also shows that the diagnosis should be accurately made and risk factors excluded before considering injection as a treatment.

## Introduction

Intra- and periarticular corticosteroid injections are widely used for the treatment of inflammatory diseases [1-4]. Amongst others, they offer significant pain relief in patients with subacromial impingement, rotator cuff tendinopathy, osteoarthritis of the acromioclavicular or glenohumeral joint, and adhesive capsulitis. However, the benefits and adverse effects of corticosteroid injections should be carefully balanced. We present the case of a young man who benefitted from the first intraarticular injection but developed a fulminant infection after the second injection.

## Case presentation

**Figure 1:**
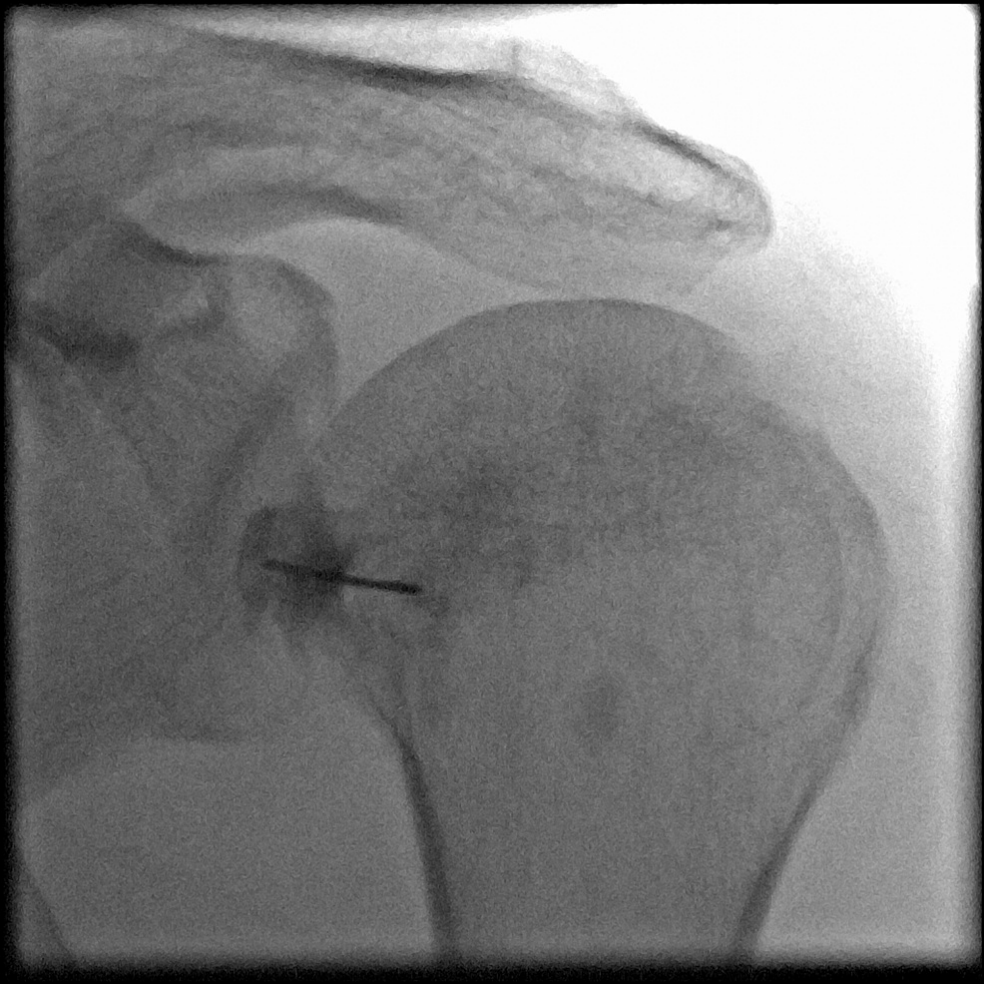
Fluoroscopy image of the left shoulder made during the first intraarticular infiltration shows the normal structure of the humeral head and the normal width of the joint space

In November 2017, a 36-year-old motorboat mechanic came to the emergency department of our hospital with intense pain in his left shoulder for one week. The symptoms had started gradually several weeks ago without preceding trauma. The clinical inspection was normal and physical examination showed a severely decreased range of motion. The body temperature was 37.5 degrees Celsius. The doctors made the diagnosis of a frozen shoulder and prescribed anti-inflammatory drugs, rest from work, and physiotherapy to improve motion. A month later, the patient presented himself in our practice with persistent pain at night and during shoulder movements. His medical history included a funnel chest operation in childhood, smoking (10 pack-years), and occasional cannabis use. Active and passive range of motion was still highly restricted with the abduction of 30°, internal rotation to the abdomen, and external rotation lacking 30° to the neutral position. Stretching the shoulder joint at the end range of motion caused rushing pain. Rotator cuff tests for internal and external rotation and supraspinatus starter function were normal. Since physiotherapy had not significantly improved motion or pain during movement in the last four weeks, we referred the patient to the department of radiology for an intraarticular corticosteroid injection. The fluoroscopy image at that time showed a normal shoulder without calcific deposits, joint space narrowing, or degenerative changes, and the diagnosis of the frozen shoulder was confirmed. An experienced radiologist performed the infiltration under sterile conditions (Figure 1).

**Figure 2:**
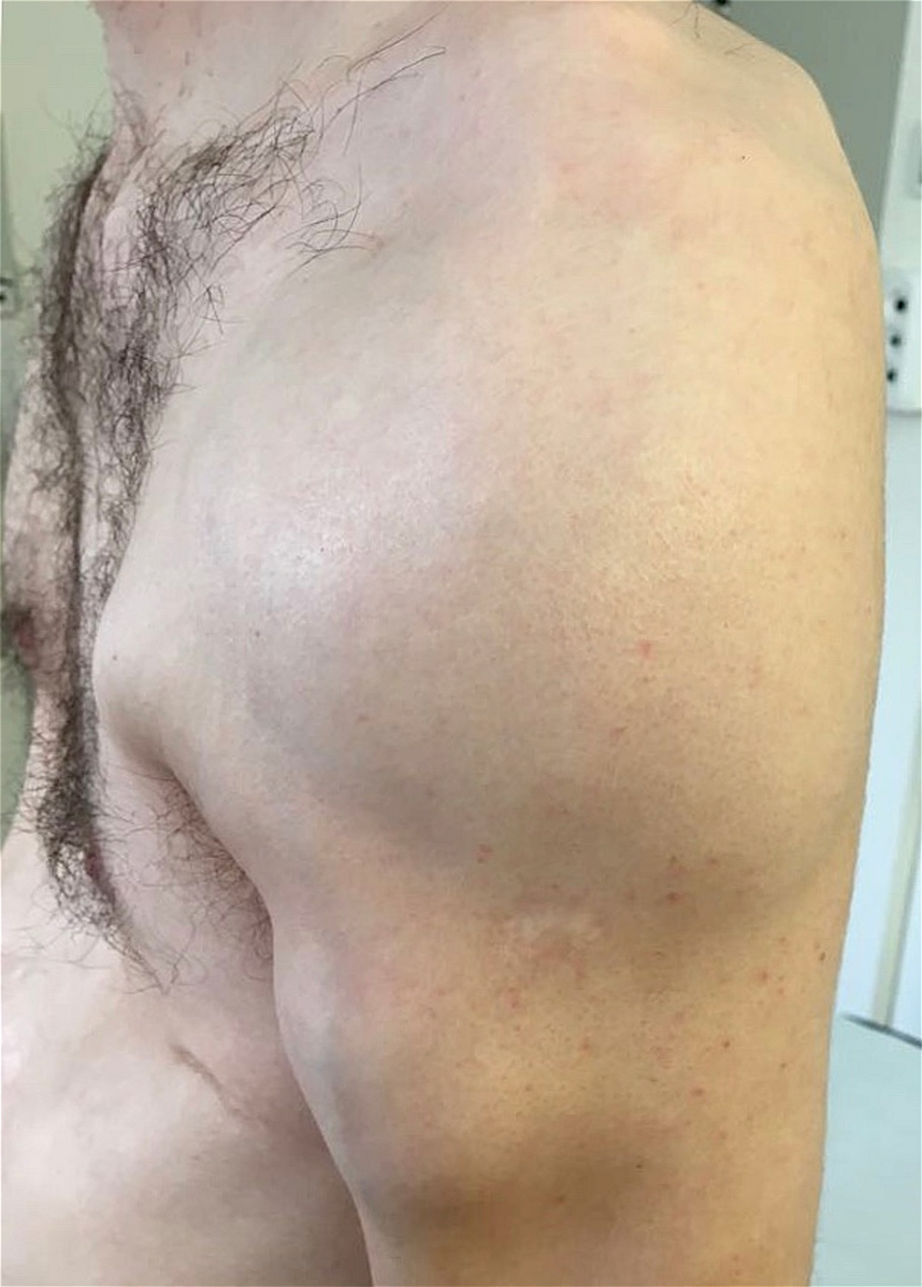
Photograph of the left shoulder taken 12 days after the second intraarticular cortisone infiltration The soft tissues are swollen; however, the skin had a normal color and temperature despite the underlying pus accumulation.

Five weeks after the corticosteroid injection, the patient reported significant pain relief for about four weeks and a slight improvement of motion. However, he had started to feel shoulder pain again one week before the follow-up visit. At his request, a second glenohumeral corticosteroid infiltration with triamcinolone 40 mg and 2 ml of bupivacaine 0.25% was made by another radiologist, again under sterile conditions, six weeks after the first injection. Twelve days later, the patient came back to the office with even more severe pain and an impressive swelling of the shoulder and the proximal part of the upper limb. The color and temperature of the skin were normal (Figure 2).

**Figure 3:**
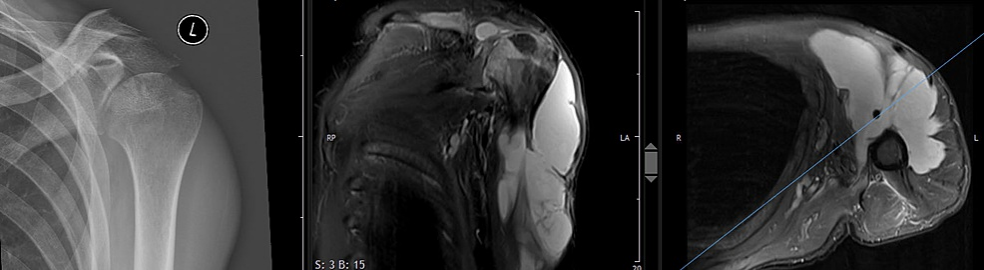
Anteroposterior radiograph (left), fat-saturated proton density-weighted coronal (middle), and axial MR image (right) of the left shoulder made 12 days after the second intraarticular cortisone infiltration A huge liquid accumulation under the thinned-out deltoid muscle, extending under the supraspinatus muscle and far distally into the upper arm, is visible. The humeral head structure is altered and the articular cartilage has disappeared.

A radiograph of the left shoulder showed joint space narrowing and an MRI of the shoulder revealed a big collection of fluid in the soft tissues of the upper arm. The images also showed an intraarticular effusion and signal alteration in the humeral head. The cartilage had disappeared (Figure 3).

The white blood cell count was 15.8 * 10^9^/L and C-reactive protein was 6 mg/L. The subdeltoid bursa was punctured and 20 ml of green pus was aspirated and sent for microbiological examination. Intravenous antibiotic treatment with amoxicillin and clavulanic acid was started. The shoulder was operated on the same day under general anesthesia. A deltopectoral approach extending to the middle of the upper arm was made. After the evacuation of the purulent liquid, multiple biopsies were taken. The wound was debrided and cleaned with an antiseptic solution. The rotator interval was opened. Inspection of the joint surfaces confirmed severe cartilage damage on both sides, the glenoid and the humeral head. The joint was abundantly irrigated. Finally, a fine-needle biopsy was taken from the humeral head in order to exclude multiple myeloma.

**Figure 4:**
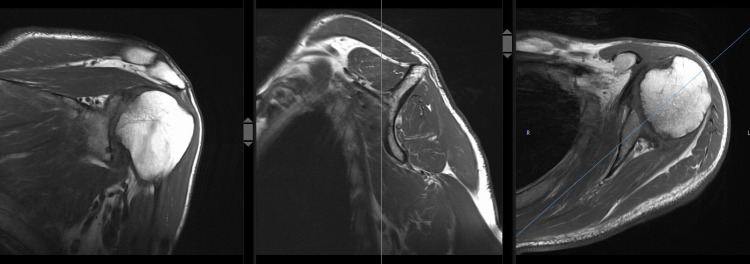
Coronal (left), sagittal (middle), and axial (right) T1-weighted turbo spin-echo MRI of the left shoulder with post-infectious arthritis, partial humeral head collapse but intact rotator cuff

The microbiologic analysis of the aspirate showed *Streptococcus pneumoniae*. Intravenous Ceftriaxone was added. Further examinations, including a chest X-ray, transthoracic echocardiography, and extended blood analyses showed no infiltration in the lungs, no valvular vegetations, and no HIV infection. Immunoglobulin levels were within the normal range and there were no signs of multiple myeloma. Following an in-depth interview, the patient mentioned gingivitis for several weeks. He was not aware of any other focus of infection. A second look was taken because of residual liquid under the deltoid and in the joint. All soft tissue probes taken during this procedure remained sterile. Two weeks after his admission, the patient could leave the hospital. Ceftriaxone and amoxicillin were administered in an outpatient setting for six weeks. Half a year after the onset of symptoms, the patient still felt pain and continued to have a stiff shoulder. New MRI showed a partial collapse of the humeral head and an intact rotator cuff (Figure 4).

**Figure 5:**
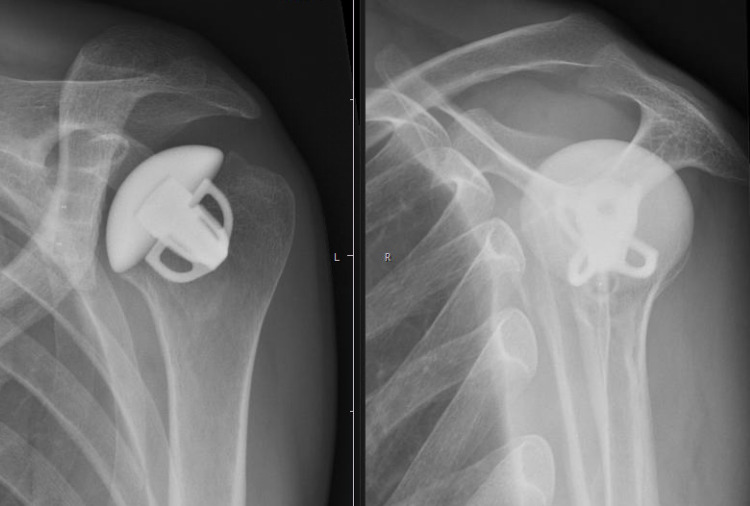
Anteroposterior radiograph (left) and Neer view (right) of the left shoulder after anatomic total shoulder replacement with a titanium short stem, a ceramic head, and a vitamin E-enhanced polyethylene glenoid component

The white blood cell count and C-reactive protein (CRP) were normal. The native joint was replaced with an anatomic total shoulder prosthesis (Affinis Short, Mathys Medical, Bettlach, Switzerland) (Figure 5).

Intraoperatively, there were no signs of persistent infection but routine biopsies revealed Propionibacterium acnes in three out of four samples. Another appropriate antibiotic treatment was therefore initiated and maintained for three months. One year after shoulder arthroplasty, the patient could resume his activity as a mechanic. At the latest follow-up four years after shoulder replacement, he reported good function and mild discomfort during overhead activities. Shoulder abduction was 160°, external rotation was 30°, and internal rotation was possible in between the scapulae. All rotator cuff tests were normal and abduction strength with the arm elevated 90° was 82 N (compared to 81 N on the other side). The absolute Constant Score was 88 points. Conventional radiographs showed no signs of prosthetic loosening.

## Discussion

Septic arthritis is a rare but severe complication of either a diagnostic or therapeutic procedure or hematogenous dissemination of pathogens from another site. In our patient, the infection appeared shortly after two intraarticular infiltrations. This might lead to the presumption that the germs were introduced into the joint by the care providers [5-7]. The fact that both radiologists strictly used aseptic techniques, that all persons in the room were equipped with masks, including the patient himself, and that the bacteria detected in the shoulder were not part of the normal skin flora makes an iatrogenic infection very unlikely. The exact cause of the infection in our patient remains therefore unclear. This is also true for the time point of bacterial colonization of the shoulder. It is conceivable that the joint was already infected at the first consultation, and that the correct diagnosis would have been septic arthritis rather than frozen shoulder. The normal clinical appearance of the shoulder in an afebrile patient, however, makes this hypothesis less probable. Determining white blood cell count and CRP before performing the second corticosteroid injection could have helped detect florid infection and should be considered in immunocompromised patients.

Pneumococcal septic arthritis is not always associated with extraarticular involvement such as pneumonia, sinusitis, otitis media, meningitis, or endocarditis [8-9]. In a literature review by Raad et al., 37 % of the patients had no overt focus of pneumococcal infection other than their septic arthritis [9]. In most cases, however, one or several underlying conditions were present. These included alcoholism, rheumatoid arthritis, HIV infection, diabetes mellitus, and steroids. Other authors made similar observations [10-12]. Our patient had no extraarticular involvement but three unfavorable conditions: gingivitis, occasional cannabis use, and two intraarticular steroid injections. Rossi et al.described the case of a 49-year-old immunocompetent man with septic arthritis involving the shoulder and knee and with spondylodiscitis due to Streptococcus pneumonia [13]. The authors supposed that the bacteria originated from the gingiva. A study on cannabis consumption and knee arthroplasty among a large US medicare population found that knee arthroplasty infection and revision rates were significantly higher in cannabis users compared to non-users [14]. This was attributed to tetrahydrocannabinols' (THCs') immunosuppressive effect [14-15]. Finally, the intraarticular applied steroids could have suppressed the local immune response. Other risk factors, such as human immunodeficiency virus [9-10,16], immunoglobulin deficiency [17-19], and multiple myeloma [10,20] were ruled out in our patient.

In most published cases, drainage and antibiotics were sufficient to treat the infection. In our patient, the presence of corticosteroids [21] and bacteria in the joint rapidly destroyed the cartilage. Therefore, anatomic total shoulder replacement became necessary. This procedure restored good shoulder function in the short term. The risk of a complication in the long term, however, is relatively high for this young mechanic. Repetitive loading of the shoulder may cause polyethylene wear and loosening of the glenoid component. In an attempt to reduce these risks, we implanted an inert ceramic head and a highly cross-linked vitamin E-blended glenoid component. This combination performed significantly better in laboratory wear tests than conventional metal on polyethylene bearings [22]. Long-term clinical results, however, are not yet available.

## Conclusions

This case highlights the importance of a detailed patient assessment before an intraarticular corticosteroid injection. In the presence of one or more risk factors, the benefit of the procedure needs to be weighed against the risk of infection. Standard radiographs should be made in order to recognize a possible pre-existing joint pathology. Finally, this case also demonstrates that septic arthritis of the shoulder can present without rubor or calor of the affected region.

## References

[1] Song A, Higgins LD, Newman J, Jain NB (2014). Glenohumeral corticosteroid injections in adhesive capsulitis: a systematic search and review. PM R.

[2] Xiao RC, Walley KC, DeAngelis JP, Ramappa AJ (2017). Corticosteroid injections for adhesive capsulitis. A review. Clin J Sport Med.

[3] Yip M, Francis AM, Roberts T, Rokito A, Zuckerman JD, Virk MS (2018). Treatment of adhesive capsulitis of the shoulder. A critical analysis review. JBJS Rev.

[4] Redler LH, Dennis ER (2019). Treatment of adhesive capsulitis of the shoulder. J Am Acad Orthop Surg.

[5] Charalambous CP, Tryfonidis M, Sadiq S, Hirst P, Paul A (2003). Septic arthritis following intra-articular steroid injection of the knee - a survey of current practice regarding antiseptic technique used during intra-articular steroid injection of the knee. Clin Rheumatol.

[6] Nallamshetty L, Buchowski JM, Nazarian LA, Narula S, Musto M, Ahn NU, Frassica FJ (2003). Septic arthritis of the hip following cortisone injection. Case report and review of the literature. J Clin Imaging.

[7] Ross K, Mehr J, Carothers B (2017). Outbreak of septic arthritis associated with intra-articular injections at an outpatient practice - New Jersey, 2017. MMWR Morb Mortal Wkly Rep.

[8] Dubost JJ, Soubrier M, De Champs C, Ristori JM, Sauvezie B (2004). Streptococcal septic arthritis in adults. A study of 55 cases with a literature review. Joint Bone Spine.

[9] Raad J, Peacock JE Jr (2004). Septic arthritis in the adult caused by Streptococcus pneumoniae: a report of 4 cases and review of the literature. Semin Arthritis Rheum.

[10] Ross JJ, Saltzman CL, Carling P, Shapiro DS (2003). Pneumococcal septic arthritis: review of 190 cases. Clin Infect Dis.

[11] Belkhir L, Rodriguez-Villalobos H, Vandercam B, Marot JC, Cornu O, Lambert M, Yombi JC (2014). Pneumococcal septic arthritis in adults: clinical analysis and review. Acta Clin Belg.

[12] Dernoncourt A, El Samad Y, Schmidt J (2019). Case studies and literature review of pneumococcal septic arthritis in adults. Emerg Infect Dis.

[13] Rossi P, Granel B, Mouly P (2010). An atypical pneumococcal arthritis. BMJ Case Rep.

[14] Law TY, Kurowicki J, Rosas S, Sabeh K, Summers S, Hubbard Z, Roche M (2018). Cannabis use increases risk for revision after total knee arthroplasty. J Long Term Eff Med Implants.

[15] Katchan V, David P, Shoenfeld Y (2016). Cannabinoids and autoimmune diseases: a systematic review. Autoimmun Rev.

[16] Mathews CW, Weston VC, Jones A, Field M, Coakley G (2010). Bacterial septic arthritis in adults. Lancet.

[17] Martinot M, Oswald L, Parisi E (2014). Immunoglobulin deficiency in patients with Streptococcus pneumoniae or Haemophilus influenzae invasive infections. Int J Infect Dis.

[18] Wang DA, Tambyah PA (2015). Septic arthritis in immunocompetent and immunosuppressed hosts. Best Pract Res Clin Rheumatol.

[19] Phuphuakrat A, Ngamjanyaporn P, Nantiruj K, Luangwedchakarn V, Malathum K (2016). Selective IgM deficiency in an adult presenting with Streptococcus pneumoniae septic arthritis. J Microbiol Immunol Infect.

[20] Riachy MA (2011). Streptococcus pneumoniae causing septic arthritis with shock and revealing multiple myeloma. BMJ Case Rep.

[21] Wernecke C, Braun HJ, Dragoo JL (2015). The effect of intra-articular corticosteroids on articular cartilage: a systematic review. Orthop J Sports Med.

[22] Alexander JJ, Bell SN, Coghlan J, Lerf R, Dallmann F (2019). The effect of vitamin E-enhanced cross-linked polyethylene on wear in shoulder arthroplasty-a wear simulator study. J Shoulder Elbow Surg.

